# Can non-pharmacological comfort care replace fentanyl in LISA? The NONA-LISA feasibility study

**DOI:** 10.1038/s41390-025-04310-8

**Published:** 2025-08-02

**Authors:** Niklas Breindahl, Tine Brink Henriksen, Christian Heiring, Emma Therese Bay, Jannie Haaber, Tenna Gladbo Salmonsen, Peter Agergaard, Emma Louise Malchau Carlsen, Martin Grønnebæk Tolsgaard, Lise Aunsholt

**Affiliations:** 1 Department of Neonatal and Pediatric Intensive Care, Copenhagen University Hospital, Rigshospitalet, Copenhagen, Denmark; 2 Department of Clinical Medicine, University of Copenhagen, Copenhagen, Denmark; 3 Prehospital Centre Region Zealand, Næstved, Denmark; 4 Department of Paediatrics and Adolescent Medicine, Aarhus University Hospital, Aarhus, Denmark; 5 Perinatal Research Unit, Department of Clinical Medicine, Aarhus University, Aarhus, Denmark; 6 Copenhagen Academy for Medical Education and Simulation (CAMES), Copenhagen University Hospital, Rigshospitalet, Copenhagen, Denmark; 7 Department of Obstetrics, Copenhagen University Hospital Rigshospitalet, Copenhagen, Denmark

## Abstract

**Background:**

Optimal comfort strategies in LISA remain unclear. This study aimed to evaluate the feasibility of a standardised approach to non-pharmacological comfort care guided by the COMFORTneo score in infants randomised to receive fentanyl or saline as premedication for LISA.

**Methods:**

Infants born before 30 weeks’ gestation were randomised to receive isotonic saline or fentanyl 0.5–1 mcg/kg intravenously as premedication for LISA. Both groups received standardised non-pharmacological comfort care. The outcomes were a modified COMFORTneo score ≥14 during the procedure, need for open-label fentanyl administration during the procedure, and inclusion rate using deferred consent. The NONA-LISA trial was registered on clinicaltrials.gov (NCT05609877).

**Results:**

From September 2023 to May 2024, 17 infants were randomised (median gestational age, 28 weeks; 77% male); eight received fentanyl and nine received saline. Modified COMFORTneo score ≥14 occurred in 4/8 (fentanyl) and 3/9 (saline), and most cases were alleviated with improved non-pharmacological comfort care only. Two infants (saline) received open-label fentanyl.

**Conclusions:**

Performing LISA with saline and standardised non-pharmacological comfort care guided by the modified COMFORTneo score was feasible. Pausing the procedure and improving non-pharmacological comfort care was sufficient in most cases with a modified COMFORTneo score ≥14 regardless of whether fentanyl or saline was used.

**Impact:**

- The Less Invasive Surfactant Administration (LISA) relies on regular spontaneous breathing to be effective. Analgesic or sedating premedication and a painful or uncomfortable stimulus can cause hypoventilation, which may prevent the LISA method from achieving its full potential.
- This feasibility study assesses if standardised non-pharmacological comfort care guided by the modified COMFORTneo score is feasible during LISA.
- Performing LISA with saline and standardised non-pharmacological comfort care guided by the modified COMFORTneo score is feasible and safe. It is possible to blind the use of premedication, and inclusion via deferred consent is feasible.

## Introduction

Neonatal respiratory distress syndrome (RDS) caused by surfactant deficiency usually develops during the first 24 h of birth despite optimised non-invasive respiratory support and remains a leading cause of mortality and morbidity in preterm infants.^1^

The concept of surfactant replacement therapy has been used for decades,^2^ and according to the European Consensus Guidelines on Management of RDS,^3^ the Less Invasive Surfactant Administration (LISA) procedure is the preferred method of surfactant treatment in spontaneously breathing infants. This procedure involves placing a thin catheter using laryngoscopy to administer surfactant intratracheally to a spontaneously breathing infant, aiming to avoid endotracheal intubation and invasive ventilation.^3–5^

The need for analgesic or other sedating premedication in LISA is debated,^6,7^ as there is a delicate balance between achieving acceptable comfort and the risk of over-sedation and hypoventilation, including apnoea and changes to the respiratory rate or tidal volumes.^8–10^ Painful or uncomfortable stimuli caused by handling the laryngoscope may also cause similar symptoms. Such complications may hinder the LISA procedure and necessitate positive pressure ventilation, potentially detrimental to the surfactant-deficient lung.^11^ In a recent study of more than 150 LISA experts from 14 countries, 41% reported no routine use of analgesic premedication,^12^ supported by other studies.^11,13,14^ However, some studies recommend the routine use of premedication,^15^ others suggest only using pharmacological agents if non-pharmacological methods are insufficient,^6,16^ or based on an individualised approach,^8,17^ and some studies prohibit premedication.^18^ While not standardised, most LISA experts use one or more non-pharmacological measures such as facilitated tucking, swaddling, or oral sucrose,^12^ which have been shown to improve procedural tolerance.^19^

We have developed a standardised approach to non-pharmacological comfort care in premature infants guided by the modified COMFORTneo score.^20^ We assessed the feasibility of using the standardised approach to non-pharmacological comfort care guided by the modified COMFORTneo score, deferred consent, and blinding in infants randomised using deferred consent to receive intravenous fentanyl 0.5–1.0 mcg/kg compared to saline as premedication for LISA as a preparation for the NONA-LISA trial (NCT05609877).

## Methods

### Trial design and ethics

The NONA-LISA trial is a multicentre, blinded, randomised controlled trial.^20^ This feasibility study included infants at two Danish level-three NICUs in Copenhagen and Aarhus before initiating the NONA-LISA trial. The NONA-LISA trial is the third neonatal trial in Denmark to obtain approval for deferred consent with an opt-out approach (The Committee on Health Research Ethics in the Region of Southern Denmark, approval number H-21078489). Written informed consent was obtained from the infant’s legal guardians as soon as possible after enrolment. The Danish Data Protection Agency approved the study protocol (approval number P-2022-594). An independent Data Management and Ethical Committee reviews the progression and safety of the NONA-LISA trial. The NONA-LISA trial was registered on clinicaltrials.gov (NCT05609877) on October 18, 2022. This manuscript conforms with the Consolidated Standards of Reporting Trials (CONSORT) Statement.^21^

### Participants

Inborn preterm infants were eligible for inclusion if they were born before 30 weeks’ gestational age and met the criteria for first-choice surfactant treatment by LISA; FiO_2_ at 0.30 and increasing while treated with continuous positive airway pressure (CPAP) ≥ 6 cm H_2_O.^3^ The primary respiratory support was CPAP in both NICUs. This study used hierarchical exclusion according to the following criteria: (1) not inborn at a study centre, (2) no need for exogenous surfactant, (3) suspicion of lung hypoplasia, (4) endotracheal intubation at any time before randomisation, (5) suspicion of pneumothorax, pulmonary haemorrhage, or pleural effusion before LISA, (6) major congenital anatomical anomalies as described by the European Surveillance of Congenital Anomalies (EUROCAT),^22^ and (7) exogenous surfactant at any time before randomisation.

### Interventions

Preterm infants received a single dose of 200 mg/kg porcine surfactant intratracheally (Curosurf®, Chiesi Pharma AB, Italy) by the LISA method and were randomised to premedication with intravenous isotonic saline (saline group) or fentanyl 0.5–1 mcg/kg (fentanyl group). All LISA procedures were performed using a dedicated surfactant catheter with a video laryngoscope and without McGills forceps.^23^ An intravenous loading dose of caffeine, 20 mg/kg, was administered.^24^ Pre- and post-procedural care, including administering atropine and naloxone, was provided based on clinical indication rather than as part of a standardised protocol.

### Non-pharmacological comfort care

One neonatologist and two neonatal nurses were present during all LISA procedures. One neonatal nurse performed the standardised approach to non-pharmacological comfort care to all infants, including light and noise reduction, swaddling, tucking, and intraoral sucrose guided by the COMFORTneo score.^20^ This approach was developed in close inter-disciplinary collaboration between neonatal nurses and neonatologists from the two participating NICUs, including team members certified as trainers and professionals according to the Newborn Individualised Developmental Care and Assessment Program (NIDCAP),^25^ the Family Infant Neurodevelopmental Education (FINE) education,^26^ and COMFORTneo score^27^ (teaching video available at https://youtu.be/I4gKTi-Jmi0 and clinical demonstration video available at https://youtu.be/7LyELgVecZ0). Building on previous evidence,^9,28^ vital signs and the modified COMFORTneo score were used before and continuously during the procedure to assess the infant’s level of discomfort or pain at baseline and continuously during the procedure.^16,20^ The modified COMFORTneo score involved all items of the original COMFORTneo score assessed continuously to avoid interrupting the procedure flow and interpreted as a binary result, using a score of ≥14 as a threshold for indicating pain or discomfort.^20^ Additionally, the infant’s respiratory drive was continuously monitored, allowing for timely stimulation in response to any signs of insufficient respiratory drive during the procedure. Open-label fentanyl could be administered at the neonatologist’s discretion by indications of a modified COMFORTneo score ≥14 despite pausing the procedure and improving non-pharmacological comfort care.^20^

### Variables

After each LISA procedure, the clinical staff completed a detailed procedure form, including procedural and feasibility data.^20^ The investigators entered baseline data from the electronic health records in the Research Electronic Data Capture (REDCap) system hosted at the Capital Region of Denmark.^29^ The data extraction template is available online.^20^ An online survey was used to gather feedback from all participating neonatologists and neonatal nurses on all intervention aspects (e.g., randomisation, non-pharmacological comfort care, and data collection). Participation in the questionnaire was anonymous and voluntary.

### Feasibility outcomes

This feasibility study evaluated the feasibility of a standardised approach to non-pharmacological comfort care guided by the COMFORTneo score for LISA, deferred consent, and blinding at two neonatal intensive care units. The clinical staff at both study sites were accustomed to administering fentanyl as premedication for LISA. This feasibility study focused on the feasibility of performing non-pharmacological comfort care guided by the modified COMFORTneo score by reporting the incidence of infants with a modified COMFORTneo score ≥14 during the procedure, the administration of open-label fentanyl, and the clinical staff’s feedback.^20^ Blinding was assessed by reporting the incidence of procedures where the clinical staff could unblind the intervention medication after the procedure, and the recruitment strategy was assessed by reporting the inclusion rates.

### Sample size

In the context of the NONA-LISA feasibility study, the investigators did not perform a sample size calculation. To ensure the timely progression of the NONA-LISA trial, the investigators scheduled the transition from the feasibility study to the NONA-LISA trial in May 2024.

### Randomisation

A person not involved in the trial constructed computer-generated random allocation sequences with permuted blocks of varying sizes of four and six to ensure proper allocation concealment.^20^ Infants were randomly assigned in a 1:1 ratio to a blue or red syringe for premedication. The randomisation sequence was stratified by trial site and gestational age at birth of less more or than 28 weeks’ gestational age.^30^

### Blinding

The investigators, the clinical staff involved in patient care, and the legal guardians were blinded to the treatment allocation. To assess the possibility of blinding the intervention, the interventions (isotonic saline vs. fentanyl) were randomly assigned to the blue or red syringe by staff not involved in patient care every 24 h. The syringes were sealed and identical in weight and appearance except for the red or blue mark. This second randomisation was performed separately and only for the feasibility study, as it could result in an unequal allocation of infants to the saline or fentanyl groups.

### Statistical methods

This feasibility study assessed the overall feasibility of conducting the NONA-LISA trial, and preliminary statistical analyses were conducted to support the evaluation of the trial’s overall feasibility. Analyses were conducted for all the randomised infants using the intention-to-treat principle. Categorical and continuous variables were presented as frequencies (counts and percentages) and using medians with interquartile ranges (IQR) due to non-Gaussian distributions. In addition to the descriptive statistics, unadjusted relative risk and 95% confidence intervals (CI) were calculated to summarise the observed data and assess the feasibility outcomes. These interpretations should be cautious due to the small sample size. All analyses were conducted using the statistical software R Studio version 4.4.3 (2025-02-28 ucrt).

## Results

### Study inclusion

**Fig. 1:**
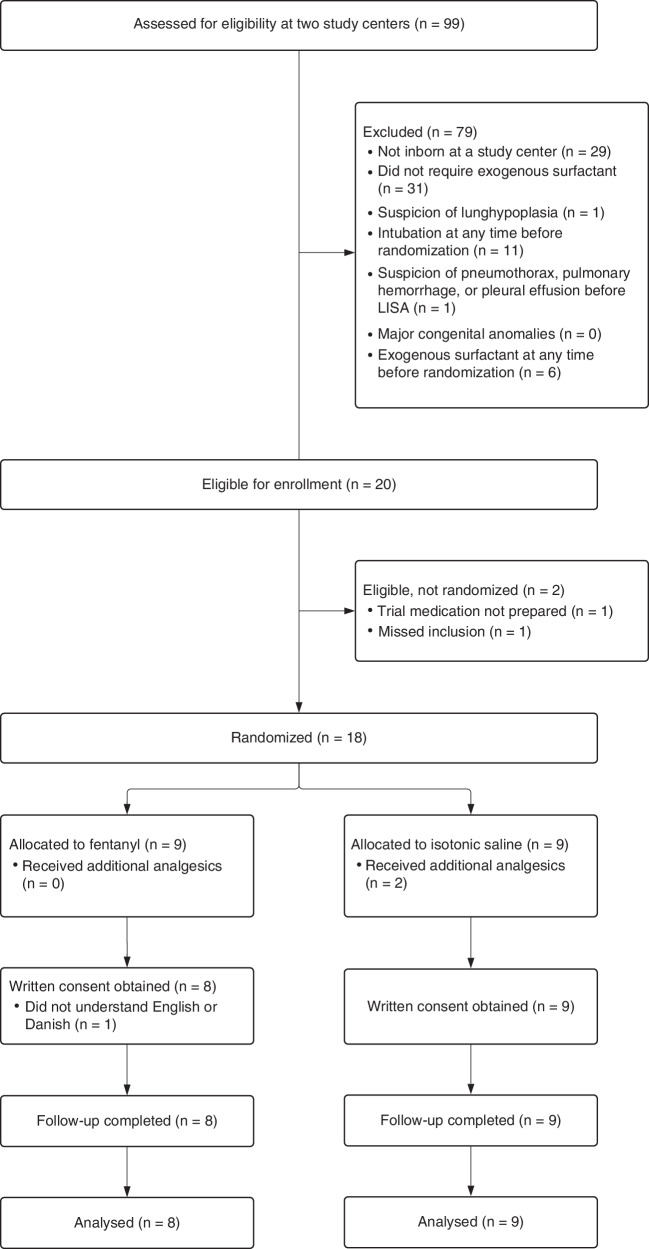
CONSORT inclusion flowchart for the NONA-LISA feasibility study. The Consolidated Standards of Reporting Trials (CONSORT) diagram of the NONA-LISA feasibility study. NONA-LISA NON-pharmacologic Approach Less Invasive Surfactant Administration, LISA Less Invasive Surfactant Administration, GA gestational age.

From September 2023 to May 2024, 99 infants born before 30 weeks’ gestational age were screened, 20 were eligible for enrolment, and 18 were randomised (nine infants were allocated to each intervention arm). Of the 79 excluded patients, 29 were not inborn at a study centre, and 31 did not require surfactant. All legal guardians, except one family, lacking proficiency in Danish or English, provided positive responses when approached by investigators following randomisation (following ethical approval for deferred consent using an opt-out approach). Data from all 17 included infants were analysed, corresponding to an inclusion rate of 85% (see Fig. 1).

### Study population

Of the infants in the total population, 77% were male (Table 1). The median [interquartile range, IQR] gestational age was 28 [26, 29] weeks, birth weight was 950 [826–1055] g, and the 5-min Apgar score was 10 [9–10].

**Table 1:** Baseline characteristics.

	Fentanyl (*N* = 8)	Saline (*N* = 9)	Total (*N* = 17)
Demographic characteristics			
Gestational age, weeks’ PMA, median [IQR]	29 [26–29]	28 [27–28]	28 [26–29]
Birth weight, g, median [IQR]	956 [838–1052]	950 [826–1055]	950 [826–1055]
Sex, *n* (%)			
Male	5 (63)	8 (89)	13 (77)
Female	3 (38)	1 (11)	4 (24)
Multiple birth, *n* (%)	3 (38)	3 (33)	6 (35)
Peripartum characteristics			
Exposure to antenatal glucocorticoids, doses prior to delivery, *n* (%)			
2 or more	6 (75)	7 (78)	13 (77)
1	2 (25)	1 (11)	3 (18)
No	0 (0)	1 (11)	1 (6)
Delivery mode, *n* (%)			
Caesarean section	6 (75)	8 (89)	14 (82)
Vaginal	2 (25)	1 (11)	3 (18)
Apgar score at 5 min, median [IQR]	10 [10–10]	10 [8–10]	10 [9–10]

Baseline characteristics stratified for saline or fentanyl as premedication for LISA and for the total population included in the NONA-LISA feasibility study.

*IQR* interquartile range, *NONA-LISA* NON-pharmacological Approach to Less Invasive Surfactant Administration.

### Procedure data

The median [interquartile range, IQR] time from birth until randomisation was 5 [2–9] h (Table 2). Nine (53%) of the LISA procedures were performed during night shifts from 19:00 to 07:00. The median [IQR] delay from the infant fulfilling the inclusion criteria to starting the procedure was 30 [28–60] min, at which time point the FiO2 was 0.38 [0.35–0.45].

**Table 2:** Procedure data.

	Fentanyl (*N* = 8)	Saline (*N* = 9)	Total (*N* = 17)
Pre-procedure characteristics			
Age at time of randomisation, h, median [IQR]	(*n* = 6) 5 [2–11]	(*n* = 9) 5 [2–8]	(*n* = 15) 5 [2–9]
Nighttime at randomisation, *n* (%)^a^	4 (50)	5 (56)	9 (53)
Delay from fulfilling the inclusion criteria until the start of the procedure, minutes, median [IQR]	30 [25–60]	38 [30–60]	30 [28–60]
nCPAP as respiratory support at randomisation, *n* (%)	8 (100)	9 (100)	17 (100)
Fio2 before the procedure, median [IQR]	0.43 [0.36–0.46]	0.35 [0.35–0.40]	0.38 [0.35–0.45]
Atropine as premedication, *n* (%)	1 (13)	0 (0)	1 (6)
Caffeine as premedication, *n* (%)	6 (75)	8 (89)	14 (82)
Total modified COMFORTneo score before the procedure, median [IQR]	(*n* = 7) 11 [10–11]	(*n* = 6) 12 [12–13]	(*n* = 13) 11 [10–12]
Procedure characteristics			
No. of laryngoscope attempts, median [IQR]	1 [1, 1]	1 [1, 2]	1 [1, 2]
Apnoea 20 s or more, *n* (%)	4 (50)	4 (44)	8 (47)
Apnoea requiring positive pressure ventilation, *n* (%)	2 (25)	0 (0)	2 (12)
Desaturation with preductal saturation <85%, *n* (%)	6 (75)	7 (78)	13 (77)
Bradycardia <100 BPM, *n* (%)	4 (50)	3 (33)	7 (41)
Observed surfactant reflux, *n* (%)	5 (62)	7 (78)	12 (71)
Naloxone to reverse apnoea, *n* (%)^b^	4 (50)	5 (56)	9 (53)
Highest modified COMFORTneo score during the procedure, median [IQR]	(*n* = 8) 14 [12–16]	(*n* = 7) 12 [11–16]	(*n* = 15) 13 [11–16]
During laryngoscopy, *n* (%)	2 (25)	7 (78)	9 (53)
During catheterisation, *n* (%)	3 (38)	0 (0)	3 (18)
During surfactant administration, *n* (%)	2 (25)	1 (11)	3 (18)
During the entire procedure, *n* (%)	1 (13)	0 (0)	1 (6)
Post-procedure characteristics			
FiO2 after the procedure, median [IQR]	(*n* = 8) 0.21 [0.21–0.29]	(*n* = 8) 0.21 [0.21–0.30]	(*n* = 16) 0.21 [0.21–0.29]

Procedure data stratified for saline or fentanyl as premedication for LISA and for the total population included in the NONA-LISA feasibility study.

*FiO2* fraction of inspired oxygen, *IQR* interquartile range, *nCPAP* nasal Continuous Positive Airway Pressure, *NONA-LISA* NON-pharmacological Approach to Less Invasive Surfactant Administration.

^a^Nighttime was between 19:00 to 07:00, and daytime was between 07:00 to 19:00.

^b^Fentanyl group: the staff expected saline in one out of four infants treated with naloxone. Saline group: the staff expected saline in all five infants treated with naloxone.

### Procedure-related complications

The most frequent procedure-related short-term complications were desaturation with preductal SpO2 < 85% and surfactant reflux (77% and 71%) (Table 2). Both groups showed similar frequencies of short-term complications (desaturation, surfactant reflux, prolonged apnoea, bradycardia, and apnoea requiring positive pressure ventilation). No serious adverse events related to the trial intervention, harms, or unintended effects were reported in either group. Naloxone was administered to 4/8 (50%) and 5/9 (56%) of infants treated with fentanyl and saline, respectively.

### Pain and discomfort during LISA

The modified COMFORTneo score immediately before the LISA procedure was high in both groups, but between eight and 13 with no clinically relevant intergroup variations (Table 2). The incidence of a modified COMFORTneo score ≥14 during the LISA procedure was 4/8 infants in the fentanyl group (50%) and 3/9 in the saline group (33%) (Table 3). The highest COMFORTneo scores were most frequently observed during laryngoscopy (Table 2). The relative risk of reaching a modified COMFORTneo score ≥14 during the LISA procedure in the fentanyl group compared to the saline group was 1.5 (95% CI: 0.47, 4.76).

**Table 3:** Feasibility outcomes.

	No./total (%)		
	Fentanyl	Saline	Relative risk (95% CI)
Modified COMFORTneo score ≥14	4/8 (50)	3/9 (33)	1.5 (0.47, 4.76)
Administration of open-label fentanyl during the procedure^a^	0/8 (0)	2/9 (22)	0.22 (0.01, 4.04)
Expected intervention true	6/8 (75)	6/9 (67)	1.13 (0.61, 2.07)

Feasibility outcomes of the NONA-LISA feasibility study, including 17 infants from two Danish neonatal intensive care units from September 2023 to May 2024.

*CI* confidence interval, *NONA-LISA* NON-pharmacological Approach to Less Invasive Surfactant Administration.

^a^Relative risk was calculated after applying a continuity correction involving adding the value ‘0.5’ to all cells in this row to avoid division by zero.

Zero infants in the fentanyl group compared to two infants in the saline group received open-label fentanyl (0.5 mcg/kg) intravenously, meaning that pausing the procedure and improving non-pharmacological comfort care was sufficient in most infants with modified COMFORTneo scores above the threshold. The relative risk of receiving open-label fentanyl in the fentanyl group compared to the saline group was 0.22 (95% CI: 0.01, 4.04).

### Blinding the use of premedication

Post-procedure, the clinical staff correctly unblinded the intervention in 12/17 infants (70%): fentanyl in 6/9 infants (67%) and saline in 6/8 infants (75%) (Table 3). The relative risk of unblinding the intervention in the fentanyl group compared to the saline group was 1.13 (95% CI: 0.61, 2.07). The clinical staff correctly indicated “saline” in all five infants in the saline group who received naloxone.

### Questionnaire outcomes

The questionnaire was completed by 15 neonatologists and seven neonatal nurses for 13 (81%) of the included infants (Table 4). A recurring theme in the feedback was the time required for non-experienced users to randomise the infant in the REDCap system, as this system demands two-factor authentication. One neonatologist commented, “It is time-consuming when quick action is needed.” The nurses requested more experience in providing the standardised approach to non-pharmacological comfort care. One neonatal nurse elaborated, “There were many disruptions - possibly because it was the first time for many of us participating in NONA-LISA. Next time, we will be better prepared and can thus optimise the non-pharmacological comfort care”.

**Table 4:** Feedback data from the clinical staff.

	*n* (%)^a^		
	Neonatologist (*N* = 15)	Neonatal nurse (*N* = 7)	Total (*N* = 22)
Responsibilities during the procedure			
Observed the intervention	1 (7)	1 (14)	2 (9)
Performed the laryngoscopy	14 (93)	0 (0)	14 (64)
Administered drugs	1 (7)	1 (14)	2 (9)
Provided non-pharmacological comfort care	2 (13)	5 (71)	7 (32)
Evaluated the modified COMFORTneo score during the procedure	1 (7)	3 (43)	4 (18)
Suggestions for improvement			
Randomisation procedure	5 (33)	0 (0)	5 (23)
Protocolized non-pharmacological comfort care	1 (7)	1 (14)	2 (9)
Drug administration	1 (7)	0 (0)	1 (5)
Modified COMFORTneo scoring	3 (20)	2 (29)	5 (23)

An online survey questionnaire was forwarded to all neonatologists and neonatal nurses who participated in the intervention. Participants answered baseline information and indicated if they had suggestions for improvement regarding any aspects of the NONA-LISA feasibility study.

*NONA-LISA* NON-pharmacological Approach to Less Invasive Surfactant Administration.

^a^A respondent with two or more responsibilities or suggestions for improvements will appear more than once in a column but only once in a row.

## Discussion

This study was designed to evaluate the feasibility of using a standardised approach to non-pharmacological comfort care guided by the COMFORTneo score in infants randomised to receive fentanyl or saline as premedication for LISA.

Previously, two smaller trials have investigated the effect of intravenous fentanyl and propofol as premedication for LISA.^9,10^ Dekker et al.^9^ randomised 78 infants to receive propofol 1 mg/kg intravenously compared to no premedication and evaluated COMFORTneo scores as the primary outcome. In these studies, COMFORTneo scores were evaluated based on video recordings of the infant post-procedure. In the propofol group, 10/42 (24%) compared to 28/36 infants (78%) in the control group had increased COMFORTneo scores, assessed post-procedure using video recordings of the infant.^9^ Both studies reported higher rates of desaturation during the LISA procedure for premedication vs. no premedication (fentanyl vs. saline: 91% vs. 69%) and need for invasive ventilation within 24 h (24% vs. 17%).^9^ The PROLISA investigates the use of propofol vs. placebo for LISA in a large sample of 542 infants and assesses procedural pain by the Faceless Acute Neonatal pain Scale (FANS), but does not consider a standardised approach to non-pharmacological treatment.^31^ In this feasibility study, 4/8 infants (50%) in the fentanyl group compared to 3/9 (33%) in the saline group had increased COMFORTneo scores. These preliminary results indicate that performing the LISA procedure with standardised non-pharmacological comfort care guided by the modified COMFORTneo score is safe and feasible. However, the wide confidence intervals suggest uncertainty around these estimates, likely resulting from the small sample size. These preliminary results should be interpreted cautiously to avoid the risk of type I and II errors. Future studies should validate the modified COMFORTneo score to guide non-pharmacological comfort care during the LISA procedure by comparing intraprocedural evaluations with blinded post-procedure evaluations based on video recordings. As a safety precaution, the NONA-LISA trial will continue to use an individualised approach to administering open-label fentanyl if pausing the procedure and improving non-pharmacological comfort care is insufficient to manage a modified COMFORTneo score ≥14 during the procedure,^20^ which is in line with previous recommendations.^3,8,16^

Blinding the use of premedication (i.e., fentanyl and saline) is crucial to avoid performance and detection bias. Other trials investigating the effect of premedication for LISA have been unblinded.^9,10^ This feasibility study demonstrated that the clinical staff was able to unblind fentanyl in 2/3 infants and saline in 3/4 infants after the procedure, which may indicate that blinding the use of premedication for LISA was not entirely possible. Importantly, the frequent use of Naloxone in both groups (50% and 56% in the fentanyl and saline groups) indicated that the blinding procedure may still effectively prevent performance bias, and that a correct guess may result from hindsight, as Naloxone administration without achieving the intended effect would indicate treatment with saline. It will be essential to monitor the blinding aspect during the NONA-LISA trial to ensure that the findings are attributable to the intervention.

Another important observation in the NONA-LISA feasibility study was the relatively high modified COMFORTneo scores in both groups immediately before the LISA procedure. Yet, the pre-procedure scores remained between eight and 13 in both groups according to the protocol.^20^ Prioritising the optimisation of comfort care before the procedure is crucial and should be effectively communicated to the clinical staff. It will be essential to monitor the modified COMFORTneo scores before the LISA procedure during the trial period, as an elevated modified COMFORTneo score before the procedure may be associated with increased pain levels during the procedure. This can potentially cause apnoea directly or indirectly through the administration of open-label fentanyl, leading to failure to maintain the concept of spontaneous breathing.

As more than half of the LISA procedures were performed during nighttime across both groups, it is paramount that the clinical staff can autonomously perform the procedure, including the standardised non-pharmacological comfort care, without the investigators' present. While some studies on LISA demonstrate the success of applying various non-pharmacological measures to complete the procedure,^9,18,19^ we suggest carefully implementing a standardised approach to non-pharmacological comfort care guided by the modified COMFORTneo score.^20^ Improving operator competence to ensure clinical proficiency in the LISA procedure is important,^16,32^ but team-based interdisciplinary LISA training may be required, even for experienced LISA operators, as these aspects are crucial for the successful performance of the LISA procedure, including non-pharmacological comfort care.^5,12^ Using the validated LISA assessment tool can improve and standardise staff training by ensuring competence in the simulated setting before advancing to supervised clinical procedures.^33^

The included infants were randomised within a median of 5 h after birth, with a median delay of 30 min from meeting the inclusion criteria. These results highlight that surfactant treatment in premature infants often needs to be initiated within a short time frame and demonstrate the importance of enrolment via deferred consent using an opt-out approach. This method of consent is accepted by regulations in the European Union and is important to secure the inclusion of sicker infants and avoid selection bias.^34,35^ Promoting this approach to unfold its potential in neonatal research remains important.^36^ The complete justification for inclusion via deferred consent is publicly available and may be used by future NONA-LISA trial sites when applying for ethical approval.^20^ Importantly, all legal guardians gave written informed consent and responded positively to the trial, supported by a review of stakeholder attitudes.^37^ One family was not proficient in Danish or English and could not provide written informed consent. The inclusion rate increased over the study period, which also served as a run-in period for the NONA-LISA trial. Increasing study awareness among the clinical staff, ensuring dedicated research staff during off-hours, and optimising the randomisation system were key elements to improve the inclusion rate. Nevertheless, continued optimisation of the inclusion rates while expanding to additional trial sites remains essential to reaching our inclusion target within a reasonable timeframe.^38^ Other key elements include adjustments to the exclusion criteria, as outborn patients should not be denied the benefits of research. The Regional Committee on Health Research Ethics approved the removal of this exclusion criterion after this study was concluded.

## Conclusion

Performing the standardised non-pharmacological comfort care guided by the modified COMFORTneo score during the LISA procedure was feasible. Similar incidence rates of modified COMFORTneo scores ≥14 were observed in the fentanyl and saline groups, and few infants required open-label fentanyl. These results indicate that a modified COMFORTneo score ≥14 was often manageable by pausing the procedure and improving non-pharmacological comfort care regardless of whether fentanyl or saline was used. Hence, we believe performing the LISA procedure with a comfort care and observation bundle with the option to pause and provide low-dose fentanyl when necessary, in case of persistent discomfort, is safe and feasible. Enrolment via deferred consent using an opt-out approach was feasible. The NONA-LISA trial is warranted to assess the potential clinical benefit of performing LISA without analgesic or sedating premedication.

## Supplementary information

CONSORT-extension-Pilot-and-Feasibility-Trials-Checklist

## Data Availability

Deidentified individual participant data (including data dictionaries, study protocols, the statistical analysis plan, and the informed consent form) will be made available upon publication to researchers who provide a methodologically sound proposal for use in achieving the goals of the approved proposal. Proposals should be submitted to lise.aunsholt@regionh.dk.
